# The Association between Untreated and Treated Hearing Loss and Cognitive Performance in Men and Women Aged 60–96 Years: Data from the Swedish “Good Aging in Skåne” Population Study

**DOI:** 10.3390/jcm13082415

**Published:** 2024-04-21

**Authors:** Paula Lundgren, Sölve Elmståhl, Henrik Ekström

**Affiliations:** The Department of Clinical Sciences in Malmö, Division of Geriatric Medicine, Skåne University Hospital, Lund University, Jan Waldenströms Gata 35, 205 02 Malmö, Sweden; paula.lundgren@hotmail.com (P.L.); solve.elmstahl@med.lu.se (S.E.)

**Keywords:** cognitive impairment, older adults, treated hearing loss, untreated hearing loss

## Abstract

**Background/Objectives**: Recent decades have witnessed a sharp increase in research investigating the association between hearing loss and cognitive impairment. Few previous studies have stratified for sex when investigating this issue, where results were inconsistent and require further clarification. Thus, the objective was to investigate the association between self-reported hearing loss and levels of cognitive impairment, stratified for sex. **Methods**: In this cross-sectional study, data were collected from 2001 to 2016. The study sample consisted of 5075 individuals, 2325 (45.8%) men, mean age 68.3 years, and 2750 (54.2%) women, mean age 70.0 years. Multiple variate ordinal regression models were constructed and adjusted for age, marital status, education, physical activity, depressive mood, hypertension, stroke, diabetes, and use of sedatives to investigate associations between groups of self-reported untreated and treated hearing loss and those reporting no hearing loss in relation to levels of cognitive impairment assessed by the Mini-Mental State Examination scale. **Results**: In men, treated hearing loss was associated with levels of cognitive impairment, odds ratio (OR) = 1.64, 95% confidence interval (CI) = 1.14–2.36. In women, both untreated hearing loss, (OR = 1.45, CI 1.07–1.98) and treated hearing loss (OR= 1.46, CI 1.06–2.04) were associated with levels of cognitive impairment. **Conclusions**: Hearing loss was found to be associated with cognitive impairment despite hearing aid use as well as awareness amongst physicians. The introduction of screening programs for hearing loss in older adults could be a crucial step for earlier identification of individuals at higher risk of developing cognitive impairment and dementia.

## 1. Introduction

With a global aging population, the prevalence of dementia continues to increase worldwide [1] and is estimated to reach 152 million in 2050 [2]. Due to the detrimental impact of dementia on the sufferer and the heavy social and economic burden it imposes on family members (and other informal caregivers), societies, and healthcare systems worldwide, dementia is considered a global health priority [3]. In the absence of a cure, current pharmacological therapy aims to minimize or, at best, delay clinical dementia-related symptoms [4]. A switch towards a more preventive strategy focusing on interventions to address potentially modifiable dementia risk factors could thus provide major public health benefits [5].

Recent decades have witnessed a sharp increase in research investigating the association between hearing loss and cognitive function. This research builds upon results from earlier research documenting a strong connection between sensory impairment (i.e., auditory and visual) and cognitive decline [6,7]. In 2017 and its follow-up in 2020, the Lancet Commission report on dementia prevention, intervention, and care identified hearing loss as the single most important potentially modifiable risk factor for dementia [4,5]. The majority of people living with hearing loss are aged over 50 years and, as with dementia, the prevalence substantially increases in line with age [8]. Almost three quarters of all individuals older than 70 years suffer from some degree of hearing loss, reaching 80% in individuals aged over 80 [9]. It has been estimated that approximately 42% of individuals with dementia and 44% of individuals with cognitive impairment have concurrent hearing loss [10]. Several longitudinal studies have shown a significant association between hearing loss and cognitive decline [11,12] and between hearing loss and incident dementia [12,13,14]. In addition, both cross-sectional and longitudinal studies have documented that hearing loss is specifically associated with a decline in global cognitive function, executive function, memory, processing speed, and attention [15,16,17,18]. In contrast, other studies have not observed any association between hearing loss and global cognitive function [19], memory [20,21], or attention [21].

The prevalence of hearing loss has been observed to be higher in men than in women [9,11,16] and men are more likely to have more severe hearing loss [12]. Furthermore, sex-related differences in cognitive function have often been observed. In older adults, women tend to outperform men in verbal learning tests [22,23] as well as tests of episodic memory and verbal fluency, whilst men tend to outperform women in tests of visuospatial ability [24,25]. Furthermore, these differences in performance are stable over time [24]. In addition, a faster rate of cognitive decline has been observed in women with mild cognitive Impairment (MCI) due to Alzheimer’s disease [26]. In contrast, the incidence of clinical sub-types of cognitive impairment has been reported to be higher in men than in women [27]. In view of this finding, surprisingly few previous studies have stratified for sex when investigating auditory–cognitive associations. Instead, most studies have used this factor as a control variable (e.g., [12,15,28]). Thus, results from previous studies stratifying for sex are scarce and even though a majority of studies have reported sex-related differences in auditory–cognitive associations [29,30,31,32], the results have been inconsistent and require further clarification.

Although evidence of the negative effect of hearing loss on cognitive function is accumulating, it remains unclear whether hearing aid use has a potential preventive effect on cognitive decline in individuals with hearing loss. Several studies have reported promising results [33,34,35], whilst others have not [11,36]. However, these studies did not stratify for sex. Consequently, it is of great interest to further elucidate the effect of hearing aid use on cognitive function and potential differences between the sexes.

Using existing data from the “Good Aging In Skåne” (GÅS) population study, including older adults aged 60–94 years, the primary purpose of this cross-sectional study is to investigate the association between cognitive impairment assessed by the Mini-Mental State Examination scale (MMSE) and self-reported hearing loss in hearing aid users as well as non-hearing aid users, stratified for sex and adjusted for age, education level, physical activity, living situation, depressive mood, hypertension, stroke, diabetes, and use of sedatives. We hypothesize that there will be gender differences in the association between hearing loss and cognitive impairment and furthermore, that the use of a hearing aid will affect the outcome, i.e., level of cognitive impairment.

## 2. Methods and Materials

### 2.1. Study Design and Population

**Figure 1:**
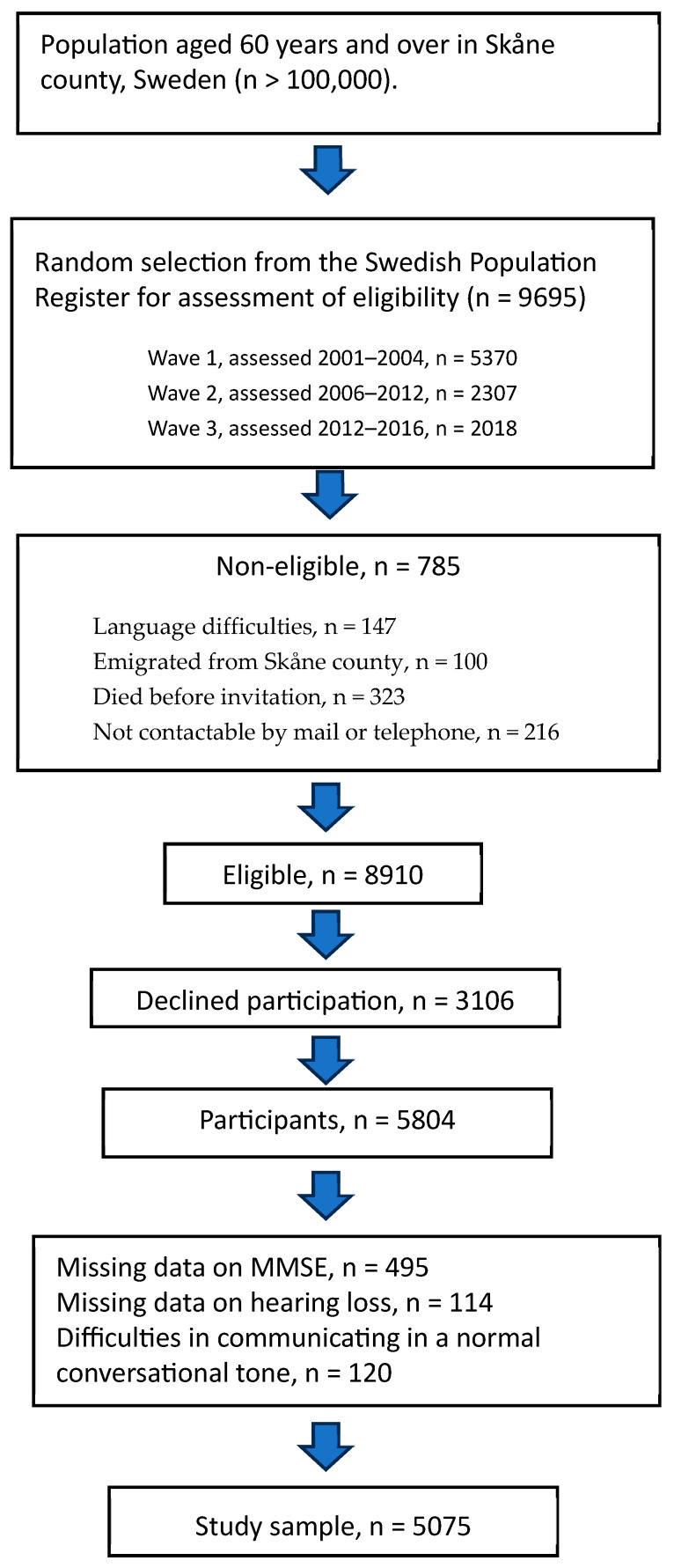
Flow chart describing the selection of the study population.

In this cross-sectional study, participants were drawn from the “Good Aging in Skåne” (GÅS) longitudinal population study, which is part of the Swedish National Aging and Care (SNAC) project [37]. The design of the GÅS study is described elsewhere [38,39]. Participants were randomized from the national population register in three waves between the years 2001 and 2016. Eligible participants were invited by phone or letter and written informed consent was obtained from those who accepted the invitation. In total, 5804 (60%) eligible participants aged 60 to 94 years living in the county of Skåne, the southernmost part of Sweden, were included. Exclusion criteria were incomplete MMSE data, which resulted in the exclusion of 495 (8.5%) participants, as well as missing hearing loss data, leading to the exclusion of a further 114 (2.0%) participants. As the MMSE was conducted in the form of an interview, another 120 individuals were excluded as they reported difficulties in communicating in a normal conversational tone. Finally, the study sample consisted of 5075 individuals, 2325 (45.8%) men and 2750 (54.1%) women (Figure 1).

### 2.2. Hearing Loss and Hearing Problems

In this study, the intention was not to objectively measure the degree of hearing loss. Instead, we wanted to explore the participants’ subjective experience of their hearing based on answers given to survey questions. By answering two yes/no questions, Do you have problems hearing? and Do you use a hearing aid? participants were divided into three groups depending on their reported hearing status; no hearing loss, hearing loss without use of hearing aid (untreated hearing loss), and hearing loss with use of hearing aid (treated hearing loss).

### 2.3. Cognitive Impairment

To assess global cognitive impairment, we used the Mini-Mental State Examination (MMSE), a short questionnaire frequently used when screening for cognitive impairment. The scale ranges from 0 to 30 points, where higher scores indicate better cognition. No cognitive impairment was set at >24 points, mild cognitive impairment at 20–24 points and moderate/severe cognitive impairment at ≤19 points [40]. We used the Swedish version of the original MMSE, which has been translated by the Swedish Association for Cognitive Disorders and is employed extensively in Sweden [41].

A trained registered nurse performed the testing. Cognitive assessment took place at the geriatric research center or in the participant’s home for health reasons.

### 2.4. Sociodemographic and Health Co-Variates

Data on education and physical activity were collected through questionnaires. Education levels were dichotomized into elementary school (9 years of compulsory studies) and high school/college (12 years), with at least 1 year of optional university studies. Physical activity was divided into sedentary (at most easier household tasks), light activities (activities 2–4 h per week, such as walks, light gardening and regular household work), and strenuous activities (physically demanding activities 1–3 h per week, such as tennis, swimming, running, or other sports) [42].

Depressive mood was assessed by the Montgomery–Åsberg Depression Rating Scale (MADRS). The MADRS includes 10 questions such as apparent sadness, inner tension, reduced appetite, and concentration difficulties. Each question is rated from 0 to 6 points and the scale ranges from 0 to 60 points. A score ≥7 points was used as a cutoff for depressive mood [43]. The MADRS has previously been validated for older adults [44]. The test was conducted as a structured interview by a psychologist.

Diseases were self-reported and confirmed by a physician in the medical examination or by reviewing medical records. Diseases included hypertension (defined as systolic blood pressure ≥ 140 and/or diastolic blood pressure ≥ 90), stroke (cerebral hemorrhage or infarction), and diabetes types 1 and 2.

Use of sedatives included drugs classified under the headings of N02 to N06 in the Anatomical Therapeutic Chemical system, i.e., analgesics, psycholeptics (sedatives, hypnotics), and psychoanaleptics (antidepressants) [45]. Data on use of sedatives were collected by a physician during the medical examination or via medical records.

### 2.5. Statistical Analysis

Sex-stratified numbers and proportions of the descriptive study population data calculated for age, cohabiting situation, education, physical activity, depressive mood, hypertension, stroke, diabetes, sedative drugs, and mild and moderate/severe cognitive impairment were tested with the chi-squared (χ^2^) test (Table 1).

Sex-stratified differences in numbers and proportions of no hearing loss, untreated hearing loss, and treated hearing loss based on age, cohabiting situation, education, physical activity, hearing problems, depressive mood, hypertension, stroke, diabetes, and use of sedative drugs were tested using the chi-squared (χ^2^) test (Table 2). Likewise, differences in numbers and proportions of cognitive impairment based on, age, cohabiting situation, education, physical activity, hearing loss, depressive mood, hypertension, stroke, diabetes, and use of sedative drugs were tested by means of the chi-squared (χ^2^) test (Table 3).

To identify associations between separate groups of hearing loss and levels of cognitive impairment in men and women, respectively, a multiple variate ordinal regression model was constructed. The associations between separate hearing loss groups and levels of cognitive impairment were adjusted for age, cohabiting situation, education, depressive mood, hypertension, stroke, diabetes, and use of sedative drugs (Table 4). In both regression models, the assumptions of non-collinearity and proportional odds were met. Only individuals with complete data were included in the regression models, which led to a loss of n = 89 (3.8%) in men and n = 149 (5.4%) in women.

No minimum or optimal sample size was calculated; instead, all eligible persons were included, n = 5075.

Statistical significance was set at a *p*-value < 0.05. All analyses were conducted using SPSS software, version 25.0 (IBM Corporation, Chicago, IL, USA).

### 2.6. Ethical Considerations

This study was conducted in accordance with the Helsinki Declaration [46] and approved by the regional ethics committee at Lund University 2010–2012, registration no. LU 744-00. All participants provided written consent and allowed retrieval of information from the National Patient Register and medical records. They were informed that they could withdraw from this study at any time.

## 3. Results

Of the 5075 eligible participants, 2325 (45.6%) were men. Men were significantly younger than women, 68.3 years (Sd 9.8) vs. 70.0 years (Sd 10.5) and were less likely to live alone (28.6% vs. 46.1%), be in a depressive mood (10.8% vs. 17.6%), and use sedative medications (6.5% vs. 10.7%) (Table 1). The prevalence of no hearing loss was 67.9% in men and 73.6% in women. Furthermore, 19.5% of the men and 14.5% of the women had an untreated hearing loss, and 12.6% of the men and 11.9% of the women had a treated hearing loss. The prevalence of mild cognitive impairment, MMSE 20–24 points, was 11.0% in men and 12.7% in women. The prevalence of moderate/severe cognitive impairment, MMSE ≤ 19 points, was 2.2% in men and 3.3% in women (Table 1).

Characteristics of the study population in relation to the different hearing groups showed that for both men and women, untreated and treated hearing loss were significantly more common in the oldest age groups, among those with lower education, diabetes, and those who suffered a stroke. In contrast to men, women reporting a depressive mood were more common in the untreated and treated hearing loss groups (Table 2).

Characteristics of the study population in relation to MMSE levels showed that older age, living alone, lower education level, low physical activity, hearing loss, depressive mood, and previous stroke increased the risk of scoring ≤ 24 or ≤19 points (Table 3). The proportion of men scoring 20–24 points on MMSE in the different hearing loss groups was as follows: no hearing loss 8.8%, untreated hearing loss 12.1%, and treated hearing loss 20.7%. The proportion of men scoring ≤ 19 points on MMSE in the different hearing loss groups was as follows: no hearing loss 1.7%, untreated hearing loss 2.4%, and treated hearing loss 4.4% (Table 3). The proportion of women scoring 20–24 points on the MMSE in the different hearing loss groups was as follows: no hearing loss 10.3%, untreated hearing loss 17.8%, and treated hearing loss 21.6%. The proportion of women scoring ≤ 19 points on the MMSE in the different hearing loss groups was as follows: no hearing loss 2.5%, untreated hearing loss 5.8%, and treated hearing loss 4.9%.

The results from the adjusted ordinal regression models showed that for men, treated hearing loss was associated with levels of cognitive impairment (OR = 1.64, CI 1.14–2.36, *p* = 0.008).

For women, both untreated hearing loss (OR = 1.45, CI 1.07–1.98, *p* = 0.017) and treated hearing loss (OR = 1.46, CI 1.06–2.04, *p* = 0.022) were associated with levels of cognitive impairment. In both men and women, age, education, and depressive mood were independently associated with levels of cognitive impairment. In women but not in men, stroke was independently associated with cognitive impairment. In men but not in women, cohabiting was independently associated with cognitive impairment (Table 4).

## 4. Discussion

By using data from the Good Aging in Skåne cohort study, the aim of the present cross-sectional study was to further investigate the association between untreated as well as treated hearing loss and cognitive impairment (measured as MMSE levels) in men and women, respectively.

The main finding of the present study was the observed sex-related differences in the association between hearing loss and cognitive impairment. In both men and women, a significant independent association between cognitive impairment and treated hearing loss was observed. However, in the untreated hearing loss group, an independent association was only observed in women. The fact that no significant relationship could be found between men with untreated hearing loss and cognitive impairment can probably be explained by the small number of men in this group and therefore lack of statistical power. In addition, the finding that hearing aids do not seem to have provided any protective effect against cognitive deterioration in either men or women is likely due to the fact that they had hearing loss for a longer period and that medical help was sought too late. It is not uncommon for older adults to wait ten years or more before seeking professional help for their hearing loss [47]. This delay can partly be explained by the stigma many people experience about their hearing loss or using hearing aids [48]. Overall, this suggests that early screening programs for hearing loss in older adults should be initiated with the aim of reducing the risk of cognitive impairment. However, by adjusting for many potential confounders, the results indicate that the connection between hearing loss and cognitive impairment cannot be fully explained by common age-related risk factors, which agrees with results from other studies adjusting for several confounders [11,13,17].

The prevalence of hearing loss was significantly higher in men than in women, as has been observed in other studies [30,49]. In addition, the prevalence of untreated hearing loss was higher in men. The prevalence of treated hearing loss was almost similar between the sexes. Both men and women with treated hearing loss were older than participants with untreated hearing loss, consistent with results from other studies [34,50]. In the present study, the use of a hearing aid could thus be regarded as an indicator of longer duration and more severe hearing loss, both of which are associated with an increased risk of dementia [6,11,12]. In men, the proportion of both mild and moderate/severe cognitive impairment was highest in hearing aid users. For mild cognitive impairment, the same was true for women. However, in contrast to men, the proportion of moderate/severe cognitive impairment (MMSE score ≤ 19 points) was higher in female non-hearing aid users (n = 23 (5.8%) vs. men, n = 11 (2.4)) and, despite the small total numbers, there is a possibility that hearing aid use might have a protective effect in women.

In general, men outperformed women on the MMSE, where the proportion of women with cognitive impairment was higher in the untreated hearing loss group. In both sexes, older age, lower education level, and depressive mood were all independently associated with cognitive impairment. At the time of this study, women were significantly older than men, were more likely to be in a depressive mood and to have a lower education level. It is well established that older age is the greatest risk factor for cognitive impairment and dementia and the risk is increased by both depression [35,51,52] and lower education level [53,54]. Considering this, differences in group characteristics could serve as an explanation for the observed variance in the association between untreated hearing loss and cognitive impairment in men and women. There is of course also the possibility that hearing loss per se has a greater negative impact on global cognitive function in women than in men. This has been indicated by results from earlier studies revealing an association between hearing loss and global cognitive decline in both cognitive intact women [31,32] and women with mild cognitive impairment [55] but not in their male counterparts. In contrast, a negative association between hearing loss, visual memory [55], and global cognitive decline [30] has been observed in men but not in women. Furthermore, Huang et al. documented a significant protective effect of hearing aid use in men but not in women [30]. Another study investigating the effect of hearing aid use on cognitive function in men and women, respectively, observed a negative association between global cognitive function, processing speed, and attention in male non-hearing aid users but not in male hearing aid users, nor in women [29]. However, the sex-related differences in the effect of hearing aid use on cognitive function in the above-mentioned studies should be interpreted with caution due to the low prevalence of hearing aid use in both studies (24 men and 6 women, respectively, 15 men and 11 women). To date, only one longitudinal study stratifying for sex has investigated the effect of hearing aid use on cognitive function in first-time hearing aid users [56]. These researchers observed a significant improvement in executive function in both sexes at the 18-month follow-up. Furthermore, in women, but not in men, a significant improvement was also observed for working memory, visual attention, and visual learning. However, it should be noted that only 33 participants were included in the analyses at follow-up and, in comparison with the general older population, the study population had a higher education level. Furthermore, women used their hearing aids to a greater extent, which could explain the sex-related difference in the effect on hearing aid use. The variability in results between studies may be due to differences in the sociodemographics of the study population (e.g., age, socioeconomics, education level, cognitive status), confounders adjusted for, measurements of hearing loss, and cognitive function.

As mentioned earlier, and in contrast to results from the above-mentioned studies, results from the present study did not demonstrate any significant protective effect of hearing aid use on cognitive impairment. Consistent with our results, other studies, although not stratifying for sex, did not observe any significant protective effect of hearing aid use on global cognitive function [11,36] or incident dementia [12,13]. In contrast, both cross-sectional [29,34,35] and longitudinal studies [33,53] have documented significant beneficial effects of hearing aid use on cognitive function as well as slower decline in episodic memory. Furthermore, some studies contained contradictory results. For example, results from a longitudinal study conducted by Brewster et al. demonstrated a protective effect of hearing aid use on executive function, language fluency, and the Boston naming test at baseline but not on global cognitive function (MMSE) or any of the cognitive tests at follow-up [16]. Due to heterogeneity in study design (i.e., cross-sectional or longitudinal) and methodology, it is difficult to draw any conclusion regarding the effect of hearing aid use on cognitive function at this point. Furthermore, the sociodemographics of the study population, measurement of hearing loss, cognitive tests administered, and confounders adjusted for have varied widely between studies.

Although beyond the scope of this article, it should be mentioned that several hypotheses have been proposed to explain the auditory–cognitive association. These have been extensively reviewed elsewhere [57,58]. In short, the common cause hypothesis proposes that hearing loss and cognitive impairment are caused by a shared neurodegenerative process in the aging brain. According to the information degradation hypothesis, more cognitive resources are allocated to auditory information processing, at the cost of other cognitive tasks. The sensory deprivation hypothesis suggests that long-term sensory deprivation negatively affects the brain, leading to permanent changes in brain structure and cognitive function. Finally, it has been proposed that the connection between hearing loss and cognitive function is mediated through social isolation and depression caused by hearing loss. However, these hypothesized pathways are not mutually exclusive, as multiple pathways can coexist and contribute to the auditory–cognitive association.

To date, results from studies investigating potential sex-related differences in auditory–cognitive associations are scarce and longitudinal studies are lacking. Despite inconsistency in results, recent research indicates that there are sex-related differences in auditory–cognitive associations. There are also potential sex-related differences in the effect of hearing aid use on cognitive function. Consequently, it is possible that sex-based health interventions are necessary to maintain quality of life in older adults. The effect of hearing aid use on cognitive function remains unclear, despite the fact that several studies have shown promising results. Identification of individuals with hearing loss and early intervention is crucial if a potential preventive effect of hearing aid use is to be achieved. Because the prevalence of undiagnosed hearing loss is high and mainly undertreated [28,47], awareness amongst physicians, together with the initiation of screening programs for hearing loss in older adults, could be an important step in earlier identification of individuals at higher risk of developing cognitive impairment and dementia [47].

### 4.1. Strengths

A large number of individuals were included in this study. By stratifying for sex, we were able to investigate potential sex-related differences between hearing loss and cognitive impairment. Furthermore, by dividing participants into three groups based on their hearing status and use of a hearing aid, we were able to study the effects of hearing aid use on cognitive impairment.

### 4.2. Limitations

As this is a cross-sectional study, it is not possible to draw any conclusions regarding causality. The difference in cognitive scores across the study population at a single time point may not reflect changes in cognition within individuals over time, nor difference in performance before and after initiation of hearing aid use. Furthermore, we do not know the duration of hearing aid use, nor how long after hearing loss our participants began using hearing aids. However, based on the age of the groups in question, it is reasonable to assume that those who report problems with hearing have had a hearing loss for a long time, probably for many years [8].

In this study, no objective measurement of hearing loss was available. However, several studies have documented that when compared to pure tone audiometry, self-reported hearing loss has sufficient sensitivity and specificity to estimate hearing loss prevalence and could be recommended for use in epidemiological studies [59,60,61]. Despite adjusting for a wide range of potential confounders, we cannot exclude residual effects of uncontrolled variables. Another limitation, which means that the results should be interpreted with caution, is that there are few among those with hearing problems who score ≤ 19 points on the MMSE.

### 4.3. Conclusions

In the present study, we observed sex-related differences in the association between untreated hearing loss and cognitive impairment. Furthermore, we found no protective effect of hearing aid use on cognitive impairment in men nor women. Our findings complement previous research investigating sex-related differences between hearing loss and cognitive impairment.

Awareness amongst physicians and initiation of screening programs for hearing loss in older adults could be a crucial step in earlier identification of individuals at higher risk of developing cognitive impairment and dementia.

Longitudinal studies with long-term follow up are required to confirm potential sex-related differences in auditory–cognitive associations and furthermore, whether hearing aid use is associated with any alteration in the rate of cognitive decline over time in men and women.

## Figures and Tables

**Table 1:** Characteristics of the study population regarding age, cohabiting situation, education, physical activity, hearing loss, depressive mood, hypertension, stroke, diabetes, sedatives, and cognitive impairment (MMSE). Significance tested with the chi-squared test, n = 5075.

Variables	Men n (%)	Missing n (%)	Women n (%)	Missing n (%)	*p*-Value
	2325 (45.8)	0 (0)	2750 (54.2)	0 (0)	
Age group					
60–69 years	1519 (65.3)	0 (0)	1616 (58.8)	0 (0)	<0.001
70–79 years	236 (10.2)		291 (10.6)		
80–89 years	507 (21.8)		701 (25.5)		
90+ years	63 (2.7)		142 (5.2)		
Age mean (Sd)	68.3 (9.8)		70.0 (10.5)		
Cohabiting situation					
Cohabiting	1691 (73.2)	16 (0.7)	1466 (53.9)	31 (1.1)	<0.001
Living alone	618 (26.8)		1253 (46.1)		
Education					
Elementary school	930 (40.2)	6 (0.3)	1293 (47.2)	13 (0.5)	<0.001
High school/college	745 (32.1)		818 (29.9)		
University	644 (27.8)		626 (22.9)		
Physical activity					
Mostly sedentary	493 (21.5)	30 (1.3)	449 (16.6)	43 (1.6)	<0.001
Light activities	1570 (68.4)		2140 (79.1)		
Strenuous activities	232 (10.1)		118 (4.4)		
Hearing loss					
No hearing loss	1578 (67.9)	0 (0.0)	2024 (73.6)	0 (0.0)	<0.001
Hearing loss, untreated	453 (19.5)		398 (14.5)		
Hearing loss, treated	294 (12.6)		328 (11.9)		
Depressive mood					
No	2025 (89.2)	54 (2.2)	2184 (82.4)	99 (3.6)	<0.001
Yes	246 (10.8)		467 (17.6)		
Hypertension					
No	967 (41.7)	8 (0.3)	1092 (39.9)	13 (0.5)	0.190
Yes	1351 (58.3)		1645 (60.1)		
Stroke					
No	2189 (94.4)	5 (0.2)	2602 (94.8)	6 (0.2)	0.459
Yes	131 (5.6)		142 (5.2)		
Diabetes					
No	2105 (90.6)	2 (0.1)	2552 (92.9)	3 (0.1)	0.003
Yes	218 (9.4)		195 (7.1)		
Sedatives					
No	2173 (93.5)	0 (0)	2455 (89.3)	0 (0)	<0.001
Yes	152 (6.5)		302 (10.7)		
MMSE, cognitive impairment					
No impairment, >24 p	2019 (86.8)	0 (0)	2310 (84.0)	0 (0)	0.008
Mild impairment, 20–24 p	255 (11.0)		350 (12.7)		
Moderate/severe impairment, ≤19 p	51 (2.2)		90 (3.3)		

**Table 2:** Sex-stratified characteristics of the different hearing groups based on age, cohabiting situation, education, physical activity, hearing problems, depressive mood, hypertension, stroke, diabetes, and sedative drugs. Significance was tested using the chi-squared test.

Variables	No Hearing Loss n (%)	Hearing Loss, Untreated n (%)	Hearing Loss, Treated n (%)	*p*-Value	No Hearing Loss n (%)	Hearing Loss, Untreated n (%)	Hearing Loss, Treated n (%)	*p*-Value
		Men, n = 2325				Women, n = 2750		
Age group								
60–69 years	1158 (76.2)	282 (18.6)	79 (5.2)	<0.001	1357 (84.0)	175 (10.8)	84 (5.2)	<0.001
70–79 years	154 (65.3)	43 (18.2)	39 (16.5)		210 (72.2)	50 (17.2)	31 (10.7)	
80–89 years	243 (47.9)	111 (21.9)	153 (30.2)		403 (57.5)	138 (19.7)	160 (22.8)	
90+ years	23 (36.5)	17 (27.0)	23 (36.5)		54 (38.0)	35 (24.6)	53 (37.3)	
Cohabiting situation								
Cohabiting	1137 (67.2)	338 (20.0)	216 (12.8)	0.613	1176 (80.2)	174 (11.9)	116 (7.9)	<0.001
Living alone	428 (69.3)	113 (18.3)	77 (12.5)		831 (66.3)	215 (17.2)	207 (16.5)	
Education								
Elementary school	587 (63.1)	206 (22.2)	137 (14.7)	0.001	884 (68.3)	225 (17.4)	184 (14.2)	<0.001
High school/college	519 (69.7)	142 (19.1)	84 (11.3)		638 (78.0)	97 (11.9)	83 (10.1)	
University	467 (72.5)	104 (16.1)	73 (11.1)		495 (79.1)	70 (11.2)	61 (9.7)	
Physical activity								
Mostly sedentary	306 (62.1)	105 (21.3)	82 (16.6)	0.001	274 (61.0)	93 (20.7)	82 (18.3)	<0.001
Light	1076 (68.5)	306 (19.5)	188 (12.0)		1619 (75.7)	283 (13.2)	238 (11.1)	
Strenuous	178 (76.7)	33 (14.2)	21 (9.1)		104 (88.1)	11 (9.3)	3 (2.5)	
Depressive mood, n (%)								
No	1394 (68.8)	394 (19.5)	237 (11.7)	0.021	1645 (75.3)	290 (13.3)	249 (11.4)	0.014
Yes	157 (63.8)	45 (18.3)	44 (17.9)		323 (69.0)	94 (19.4)	63 (13.5)	
Hypertension, n (%)								
No	664 (68.7)	187 (19.3)	116 (12.0)	0.713	841 (77.0)	146 (13.4)	105 (9.6)	0.002
Yes	911 (67.9)	263 (19.5)	177 (13.1)		1173 (71.3)	252 (15.3)	220 (13.4)	
Stroke, n (%)								
No	1507 (68.8)	417 (19.0)	265 (12.1)	<0.001	1927 (74.1)	373 (14.3)	302 (11.6)	0.019
Yes	70 (54.4)	34 (26.0)	27 (20.6)		91 (64.1)	25 (17.6)	26 (18.3)	
Diabetes, n (%)								
No	1442 (68.5)	405 (19.2)	258 (12.3)	0.118	1899 (74.4)	356 (13.9)	297 (11.6)	0.001
Yes	136 (62.4)	46 (21.1)	36 (16.5)		122 (62.6)	42 (21.5)	31 (15.9)	
Sedatives, n (%)								
No	1481 (68.2)	427 (19.7)	265 (12.2)	0.045	1819 (74.1)	357 (14.5)	279 (11.4)	0.032
Yes	97 (63.8)	26 (17.1)	29 (19.1)		205 (69.5)	41 (13.9)	49 (16.9)	

**Table 3:** Comparisons of levels of MMSE, 25–30 points, 20–24 points and ≤19 points, based on age, cohabiting situation, education, physical activity, hearing loss, depressive mood, hypertension, stroke, diabetes, and use of sedatives. Significance tested with the chi-squared test.

Variables		Men n = 2325				Women n = 2750		
	25–30 p	20–24 p	≤19 p	*p*-value	25–30 p	20–24 p	≤19 p	*p*-value
Age group								
60–69 years	1410 (92.5)	94 (6.2)	15 (1.0)	<0.001	1477 (91.4)	118 (7.3)	21 (1.2)	<0.001
70–79 years	198 (83.9)	28 (11.9)	10 (4.2)		239 (82.1)	46 (15.8)	6 (2.1)	
80–89 years	369 (72.8)	116 (22.9)	22 (4.3)		519 (74.0)	145 (20.7)	37 (5.3)	
90+ years	42 (66.7)	17 (27.0)	4 (6.3)		75 (52.8)	41 (28.9)	26 (18.3)	
Cohabiting situation								
Cohabiting	1497 (88.5)	165 (9.8)	29 (1.7)	0.001	1287 (87.8)	145 (9.9)	34 (2.3)	<0.001
Living alone	513 (83.0)	84 (13.6)	21 (3.4)		1010 (80.6)	185 (15.6)	48 (3.8)	
Education								
Elementary school	746 (80.2)	148 (15.9)	36 (3.9)	<0.001	1001 (77.4)	223 (17.2)	69 (5.3)	<0.001
High school/college	665 (87.9)	77 (10.3)	13 (1.7)		713 (87.2)	92 (11.2)	13 (1.6)	
University	616 (95.7)	26 (4.0)	2 (0.3)		591 (94.4)	32 (5.1)	3 (0.5)	
Physical activity								
Mostly sedentary	375 (76.1)	92 (18.7)	26 (5.3)	<0.001	296 (65.9)	105 (23.4)	48 (10.7)	<0.001
Light activity	1416 (90.2)	133 (8.5)	21 (1.3)		1886 (88.1)	222 (10.4)	32 (1.5)	
Strenuous activity	211 (90.9)	19 (8.2)	2 (0.9)		110 (93.2)	7 (5.9)	1 (0.8)	
Hearing loss								
No	1412 (89.5)	139 (8.8)	27 (1.7)	<0.001	1765 (87.2)	208 (10.3)	51 (2.5)	<0.001
Yes, untreated	387 (85.4)	55 (12.1)	11 (2.4)		304 (76.4)	71 (17.8)	23 (5.8)	
Yes, treated	220 (74.8)	61 (20.7)	13 (4.4)		241 (73.5)	71 (21.6)	16 (4.9)	
Depressive mood								
No	1795 (88.6)	197 (9.7)	33 (1.6)	<0.001	1902 (87.1)	234 (10.7)	48 (2.2)	<0.001
Yes	192 (78.0)	43 (17.5)	11 (4.5)		352 (75.4)	93 (19.9)	22 (4.7)	
Hypertension								
No	838 (87.1)	106 (11.0)	23 (2.4)	0.755	952 (87.3)	103 (9.4)	36 (3.3)	<0.001
Yes	1177 (87.1)	148 (11.0)	26 (1.9)		1348 (84.1)	245 (14.9)	52 (3.2)	
Stroke								
No	1916 (87.5)	230 (10.5)	43 (2.0)	<0.001	2216 (85.2)	310 (11.9)	76 (2.9)	<0.001
Yes	100 (76.3)	24 (18.3)	7 (5.3)		90 (63.4)	39 (27.5)	13 (9.2)	
Diabetes								
No	1842 (87.5)	223 (10.6)	40 (1.9)	0.007	2153 (84.4)	318 (12.5)	81 (3.2)	0.201
Yes	177 (81.2)	31 (14.2)	10 (4.6)		155 (79.5)	32 (16.4)	8 (4.1)	
Sedatives								
No	1890 (87.0)	235 (10.8)	48 (2.2)	0.664	2064 (84.1)	307 (12.5)	84 (3.4)	0.293
Yes	129 (84.9)	20 (13.2)	3 (2.0)		246 (83.4)	44 (14.6)	6 (2.0)	

**Table 4:** Sex-stratified results of multiple ordinal regressions with MMSE (trichotomized at cutoff ≤24 points and ≤19 points) as dependent variable and age, cohabiting situation, education, physical activity, hearing loss, depressive mood, hypertension, stroke, diabetes, and use of sedatives as independent variables.

Independent Variables	Men n = 2236				Women n = 2601			
	Estimate	*p*-value	OR ^a^	95% CI ^b^	Estimate	*p*-value	OR ^a^	95% CI ^b^
Age cohort								
(ref. age cohort 60 years) ^c^								
70	0.664	0.003	1.94	1.26–2.99	0.444	0.024	1.56	1.06–2.29
80	1.106	<0.001	3.02	2.18–4.20	0.733	<0.001	2.08	1.53–2.81
90	1.332	<0.001	3.78	1.99–7.21	1.433	<0.001	4.19	2.59–6.86
Cohabiting situation								
(ref. living alone) ^c^								
Cohabiting	−0.387	0.009	0.68	0.51–0.91	0.169	0.192	1.18	0.92–1.52
Education								
(ref. elementary school) ^c^								
High school	−0.511	0.001	0.60	0.44–0.81	−0.346	0.014	0.71	0.53–0.93
University	−1.442	<0.001	0.24	0.15–0.37	−1.104	<0.001	0.33	0.21–0.50
Physical activity								
(ref. sedentary) ^c^								
Light	−0.693	<0.001	0.50	0.37–0.67	−0.789	<0.001	0.45	0.35–0.59
Strenuous	−0.380	0.163	0.68	0.40–1.17	−0.766	0.057	0.46	0.21–1.02
Hearing loss								
(ref. no hearing loss) ^c^								
Hearing loss, untreated	0.174	0.322	1.19	0.84–1.68	0.377	0.017	1.45	1.07–1.98
Hearing loss, treated	0.496	0.008	1.64	1.14–2.36	0.385	0.022	1.46	1.06–2.04
Depressive mood	0.636	<0.001	1.89	1.31–2.71	0.607	<0.001	1.83	1.40–2.40
(ref. no dep. mood) ^c^								
Hypertension	−0.167	0.235	0.85	0.64–1.11	0.199	0.129	1.22	0.94–1.58
(ref. no hypertension) ^c^								
Stroke	0.009	0.973	1.01	0.61–1.66	0.533	0.014	1.70	1.12–2.60
(ref. no stroke) ^c^								
Diabetes	−0.219	0.304	0.80	0.52–1.22	−0.151	0.464	0.86	0.57–1.29
(ref. no diabetes) ^c^								
Sedatives	−0.227	0.404	0.79	0.46–1.36	−0.152	0.425	0.86	0.5–1.25
(ref. no sedatives) ^c^								

^a^ OR = odds ratio, ^b^ CI = confidence interval, ^c^ ref. = reference category.

## Data Availability

The authors confirm that the data supporting the findings of this study are available in the article.
